# Homotopy perturbation method with Laplace Transform (LT-HPM) for solving Lane–Emden type differential equations (LETDEs)

**DOI:** 10.1186/s40064-016-3487-4

**Published:** 2016-10-22

**Authors:** Rajnee Tripathi, Hradyesh Kumar Mishra

**Affiliations:** Department of Mathematics, Jaypee University of Engineering and Technology, Guna, MP 473226 India

**Keywords:** Homotopy Perturbation Method (HPM), Laplace Transform (LT), Singular Initial value problems (IVPs), Lane–Emden type equations

## Abstract

In this communication, we describe the Homotopy Perturbation Method with Laplace Transform (LT-HPM), which is used to solve the Lane–Emden type differential equations. It’s very difficult to solve numerically the Lane–Emden types of the differential equation. Here we implemented this method for two linear homogeneous, two linear nonhomogeneous, and four nonlinear homogeneous Lane–Emden type differential equations and use their appropriate comparisons with exact solutions. In the current study, some examples are better than other existing methods with their nearer results in the form of power series. The Laplace transform used to accelerate the convergence of power series and the results are shown in the tables and graphs which have good agreement with the other existing method in the literature. The results show that LT-HPM is very effective and easy to implement.

## Background

Two astrophysicists, Jonathan Homer Lane and Robert had explained the Lane–Emden type differential equations. In this study, they had designed these types of differential equations, which is a dimensionless structure of Poisson’s equation for the gravitational potential of a self-gravitating, spherically symmetric, polytropic fluid and the thermal behavior of a spherical bunch of gas according to the laws of thermodynamics (Lane 1870; Richardson 1921). The Lane–Emden type of differential equation is also called the polytropic differential equations and it is given by:

$$\frac{1}{{\tau^{2} }}\frac{d}{d\tau }\left( {\tau^{2} \frac{d\varGamma }{d\tau }} \right) + \varGamma^{n} = 0$$

where $\tau$ is a dimensionless radius and $\varGamma$ is linked to the density (and accordingly the pressure) by $\rho = \rho_{c} \tau^{n}$ for central density $\rho_{c}$. The index n is the polytropic index to make easy in the form of polytropic equation of state, $P = K\rho^{{1 + \frac{1}{n}}}$, where P and $\rho$ are the pressure and K density, respectively, and n is a constant of proportionality.

Boundary conditions are

$$\tau (0) = 1,\quad \tau^{{\prime }} (0) = 0.$$

Thus, the solutions describe the gallop of pressure and density (with radius), which is known as polytropes of n.

The Lane–Emden equation has been useful to model some phenomena in astrophysics and mathematical physics such as the principle of stellar structure, the thermal nature of the spherical bunch of the gas, isothermal gas spheres (IGSs), and the principle of thermionic currents (Wazwaz 2011). According to the extensive study of many physicists, these equations have been applicable in the case of astrophysics such as kinetics of combustion and the Landau–Ginzburg major phenomenon (Dixon and Tuszynski 1990; Fermi 1927; Fowler 1930; Frank-Kamenetskii 1921). The numerical solutions of the Lane–Emden equations [LEes] are very difficult due to the expressive nature of the nonlinearities term. Therefore, much attention has been applied to the better and more powerful methods for establishing a solution, approximate or exact, analytical or numerical, to the Lane–Emden equations (LEes).

Recently many analytical techniques have been used for the solution of Lane–Emden type equation, for example, Hosseini and Abbasbandy (2015) described the hybrid Spectral Adomain Decomposition Method for solving Lane–Emden type of differential equations by combining the spectral method and Adomain Decomposition method. Since the description of this method is very long and difficult for solving these types of numerical problems. Our method is suitable and best to determine these results, Modified Laplace decomposition method for Lane–Emden type differential equations by Yin et al. (2013), A new algorithm for solving singular IVPs of Lane–Emden type differential equation by Motsa and Sibanda (2010). The author has solved the Lane–Emden type equations by Successive Linearization Method Since it is a very complicated method to get the solution of these types of problem in terms of an exact solution. Various methods for Lane–Emden equations have described by some authors in Rafiq et al. (2009), Baranwal et al. (2012), Liao (2003), Shawagfeh (1993), Wazwaz (2001), A new method for solving singular IVPs in the second order ordinary differential equations by Wazwaz (2001), Nouh (2004), Romas (2003) and other researchers have been studied several methods to attempt nonlinear problems. These methods have also been successfully applied to, analytical solution of convection–diffusion problem by combining Laplace transform method and homotopy perturbation method by Gupta et al. (2015), Mandelzweig and Quasi (2001), Singh et al. (2012), Nazari-Golshan et al. (2013), Explicit solution of Helmholtz equation and sixth-order KdV equation by Rafei and Ganji (2006), Ganji and Rajabi (2006), Jang (2016), Exact solutions of some coupled nonlinear partial differential equations(NPDE) using the homotopy perturbation method by Sweilam and Khader (2009), Homotopy perturbation method for solving viral dynamical model (VDM) by Merdan and Khaniyev (2010), nonlinear population dynamics models(NPDMs) by Chowdhury and Hashim (2007, Hashim and Chowdhury (2007), A new dispersion-relation preserving method for integrating the classical boussinesq equation by Jang (2017), The modified homotopy perturbation method (MHPM) for solving strongly nonlinear oscillators by Momani et al. (2009), Pandit (2014) and pure nonlinear differential by Cveticanin (2006), Inverse problem (IP) of diffusion equation by He’s homotopy perturbation method by Shakeri and Dehghan (2007), A Higher order Numerical Scheme for singularly perturbed Burger–Huxley equation by Jiwari and Mittal (2011).

The Laplace transform is a superb technique for solving linear and nonlinear Lane–Emden type differential equation and has enjoyed much success in the field of science and engineering. On the other hand, Laplace Transform (LT) has played an important role in mathematics (Spiegel and Teoríay 1988), not only for its theoretical interest but also because such method allows solving, in a simpler fashion, many problems in the realm of science, in comparison with other mathematical techniques. It is totally difficult to solve nonlinear equations because of the problems caused by nonlinear terms. Homotopy perturbation technique by He (1999), the homotopy perturbation method using Laplace Transform by Madani et al. (2011; Abbasbandy (2006); Gupta and Gupta 2011), a numerical solution of two-point boundary value problems using Galerkin-Finite element method by Sharma et al. (2012) have solved nonlinear problems. The Homotopy perturbation methods with Laplace transform (LT-HPM) and other methods have external significant thought in the literature. Moreover, The Homotopy Perturbation Method (HPM) by He (1999a, b, 2003) and the Variational Iteration Method (VIM) (Khuri and Sayfy 2012) are combined with the Laplace transform (LT) to develop a more effective technique for handling many nonlinear problems. A comparative study of model of matrix and finite elements methods for two-point boundary value problems is given by Sharma et al. (2012).

In the present paper, a Homotopy Perturbation Method (HPM) with Laplace Transform (LT) to solve the general type of Lane–Emden differential equations is proposed; the paper is organized as follows: The Homotopy Perturbation method is given in “Preliminaries” section. The Lane–Emden Equations (LEes) is given in “The Land–Emden equation” section. Homotopy Perturbation Method with Laplace Transform (LT-HPM) is given in “Homotopy perturbation method” section. Some examples of a different kind are given in “Results and discussion” section. Finally, the conclusion is explained in “Conclusions” section.

## Preliminaries

### Homotopy perturbation method

Consider the nonlinear differential equation

1 $$A(\varGamma ) - \zeta (r) = 0,\quad r \in \varOmega ,$$

with the boundary conditions of

2 $$B\left( {\varGamma ,\frac{\partial \varGamma }{\partial n}} \right) = 0,\quad r \in \chi .$$

where A, B, $\zeta (r)$ and $\chi$ are a general differential operator, a boundary operator, a known analytic function and the boundary of the domain Ω, respectively and $\frac{\partial \varGamma }{\partial n}$ denotes the differentiation of $\varGamma$ with respect to $n$.

We can distribute the operator A into a linear part K and a nonlinear part N. Equation (1) may possibly be written as:

3 $$K(\varGamma ) + N(\varGamma ) - \zeta (r) = 0.$$

By the Homotopy technique, we construct a Homotopy $v(r,p):\varOmega \times [0,1] \to R$ which satisfies:

$$\begin{aligned} & H(v,p) = (1 - p)[K(v) - K(\varGamma_{0} )] + p[A(v) - \zeta (r)] = 0, \\ & or \\ & H(v,p) = K(v) - K(\varGamma_{0} ) + pK(\varGamma_{0} ) + p[N(v) - \zeta (r)] = 0, \\ \end{aligned}$$

*p*∈ [0, 1] is an embedding parameter, while $\varGamma_{0}$ is an initial approximation which satisfies the boundary conditions. Obviously, from above equations, we will have

4 $$H(v,0) = K(v) - K(\varGamma_{0} ) = 0.$$

5 $$H(v,1) = A(v) - \zeta (r) = 0.$$

Now changing the process of *p* from 0 to 1 is even-handed that of $v(r,p)$ from $\varGamma_{0}$ to $\varGamma (r)$. According to the concept of topology, this is called deformation, whereas $K(v) - K(\varGamma_{0} )$ and $A(v) - \zeta (r)$ are called homotopy. If we consider the embedding parameter *p* is a minor parameter, applying the classical perturbation technique, we can assume that the solution of Eqs. (4) and (5) can be defined as a power series in *p:*

6 $$v = v_{0} + v_{1} p + v_{2} p^{2} + v_{3} p^{3} + \cdots \cdots \infty ,$$

putting p = 1 in Eq. (6), we have

7 $$\varGamma = \mathop {\lim }\limits_{p \to 1} v = v_{0} + v_{1} + v_{2} + v_{3} + \cdots \cdots .$$

The coupling of the perturbation method and the homotopy method is said to be HPM. The series (6) is convergent for most cases. However, the convergent rate depends on the nonlinear operator $A(v)$. Moreover, He (1999) made the following suggestions:

1. The second derivative of $N(v)$ with respect to *v* must be minor because the parameter may be comparatively large, i.e. *p* → 1.
2. The norm of $K^{ - 1} \left( {\frac{\partial N}{\partial v}} \right)$ must be lesser than one so that the series converges.

### Laplace transform method

#### **Definition**

The Laplace transform of a function ξ(τ), is defined by

$$\xi (s) = L[\xi (\tau )] = \int\limits_{0}^{\infty } {e^{ - s\tau } } \xi (\tau )d\tau ;\quad \tau \ge 0$$

(Whenever integral on RHS exists) where, $\tau$ ≥ 0, s is real and L is the Laplace transform operator.

## The Lane–Emden equation

Lane–Emden type differential equations are singular initial value problems (IVPs) describing the second order homogeneous and nonhomogeneous linear and nonlinear differential equations which have been applicable in the many fields. The mathematical representation of Lane–Emden equation is:

8 $$\varGamma^{\prime \prime } + \frac{2}{\tau }\varGamma^{\prime } + \zeta (\varGamma ) = 0,\quad 0 \le \tau \le 1,$$

subject to conditions,

9 $$\varGamma (0) = A,\quad \varGamma^{{\prime }} (0) = B.$$

where A and B are constants $\zeta (\varGamma )$ is a real-valued continuous function.

These types of equations generally occur in the principle of stellar structure, the thermal behaviour of a spherical bunch of gas, isothermal gas spheres (IGSs) and the principle of thermionic currents (Richardson 1921; Chandrasekhar 1967; Davis 1962).

On the other hand, A nonlinear class of singular initial value problems of Lane–Emden type has the following form:

10 $$\varGamma^{{\prime \prime }} + \frac{2}{\tau }\varGamma^{{\prime }} + \zeta (\tau ,\varGamma ) = \varphi (\tau ),\quad 0 \le \tau \le 1$$

The solution of the Lane–Emden type differential equation is numerically challenging because of the singularity behaviour at the origin. The solutions of the Lane–Emden equation were given by Wazwaz (2001), Shawagfeh (1993), Mishra (2014), the homotopy perturbation method (HPM) of above type by Davis (1962), Yildrim and Ozis (2007), He (2003), 2006), Ramos (2008), Exact solution of Generalized Lane–Emden equation is given by Goenner and Havas (2000). The major advantage of this method is its capability of combining the two powerful methods to obtain exact solutions of nonlinear equations. Therefore, Homotopy Perturbation Method using Laplace transform (LT-HPM) accelerates the rapid convergence of the series solution. In this paper, we will apply the (LT-HPM) to obtain exact or approximate analytical solutions of the Lane–Emden type equations.

## Homotopy perturbation method with laplace transform (LT-HPM)

In this section, we will briefly discuss the use of the LT-HPM for the solution of Lane–Emden equation given in “The Land–Emden equation” section, consider the following:

11 $$\varGamma^{{\prime \prime }} + \frac{2}{\tau }\varGamma^{{\prime }} + \zeta (\tau ,\varGamma ) = \varphi (\tau ),\quad 0 \le \tau \le 1.$$

Multiplying $\tau$ and then taking the Laplace transform on both sides of (11) we get:

12 $$- s^{2} L^{{\prime }} (\varGamma ) - \varGamma (0) + L\{ \tau \zeta (\tau ,\varGamma ) - \tau \varphi (\tau )\} = 0,$$

where *L* is the operator of Laplace transform and $L'(\varGamma ) = \frac{dL(\varGamma )}{ds}$

13 $$L^{{\prime }} \left( \varGamma \right) = - s^{ - 2} \varGamma (0) + s^{ - 2} L[\tau \zeta (\tau ,\varGamma ) - \varphi (\tau )].$$

by integrating both sides of (13) with respect to s, we have

14 $$L(\varGamma ) = - \int {s^{ - 2} \varGamma (0)} ds + \int {s^{ - 2} } L[\tau \zeta (\tau ,\varGamma ) - \tau \varphi (\tau )]ds.$$

taking inverse Laplace transform on both sides of (14), we get

15 $$\varGamma (\tau ) = - L^{ - 1} \left\{ {\int {\left( {s^{ - 2} \varGamma (0)} \right)ds} } \right\} + L^{ - 1} \left\{ {\int {s^{ - 2} L[\tau \zeta (\tau ,\varGamma ) - \tau \varphi (\tau )]} ds} \right\}.$$

By using initial condition (9), we have

16 $$\varGamma (\tau ) = A + L^{ - 1} \left\{ {\int {s^{ - 2} L[\tau \zeta (\tau ,\varGamma ) - \tau \varphi (\tau )]ds} } \right\}.$$

We decompose $\zeta (\varGamma ,\tau )$ into two parts

17 $$\zeta (\tau ,\varGamma ) = K[\varGamma (\tau )] + N[\varGamma (\tau )].$$

where $K[\varGamma (\tau )]$ and $N[\varGamma (\tau )]$ denote the linear term and the nonlinear term respectively. The Homotopy perturbation method and the He’s polynomials can be used to handle Eq. (12) and to address the nonlinear term. LT-HPM defines a solution by an infinite series of components given by:

18 $$\varGamma (\tau ) = \sum\limits_{n = 0}^{\infty } {p^{n} } \varGamma_{n} (\tau ).$$

where the terms $\varGamma_{n} (\tau )$ are to recursively calculate and the nonlinear term $\zeta$ ($\varGamma$) can be given as

19 $$N(\varGamma ) = \sum\limits_{n = 0}^{\infty } {p^{n} } H_{n} (\varGamma ).$$

where $N(\varGamma )$ is a non-linear term and $H_{n} (\varGamma )$ is He’s polynomial.

For some He’s polynomial $H_{n}$ (Mishra 2012) that are given by

20 $$H_{n} (\varGamma_{0} ,\varGamma_{1} ,\varGamma_{2} \ldots \varGamma_{n} ) = \frac{1}{n!}\frac{{\partial^{n} }}{{\partial p^{n} }}\left[ {N\left( {\sum\limits_{n = 0}^{\infty } {p^{i} \varGamma_{i} } } \right)} \right]_{p = 0} \quad n = 0,1,2, \ldots .$$

Substituting the value of (19) and (20) in (18), we get

21 $$\sum\limits_{n = 0}^{\infty } {p^{n} } \varGamma_{n} = A + p\left\{ {L^{ - 1} \int\limits_{0}^{s} {s^{ - 2} } \left\{ {\left( {L\left( {\left( {\sum\limits_{n = 0}^{\infty } {p^{n} H_{n} (\tau )} } \right) - \tau \left( {\sum\limits_{n = 0}^{\infty } {p^{n} } \varGamma_{n} (\varGamma )} \right)} \right)} \right)} \right\}ds} \right\}.$$

which is the coupling of the Laplace transformation and the Homotopy Perturbation Method (LT-HPM) using He’s polynomials by Mishra (2012, 2014). Comparing the coefficient of like powers of p, the following approximations are obtain

22 $$\left. \begin{aligned} p^{0} :\varGamma_{0} (x) & = A, \\ p^{1} :\varGamma_{1} (\tau ) & = - L^{ - 1} \left[ {\int {s^{ - 2} \left( {\left( {L\left( {\tau \left( {H{}_{0}} \right) - \tau (\varGamma_{0} )} \right)} \right)} \right)} ds} \right], \\ p^{2} :\varGamma_{2} (\tau ) & = - L^{ - 1} \left[ {\int {s^{ - 2} \left( {\left( {L\left( {\tau \left( {H{}_{1}} \right) - \tau (\varGamma_{1} )} \right)} \right)} \right)} ds} \right], \\ p^{3} :\varGamma_{3} (\tau ) & = - L^{ - 1} \left[ {\int {s^{ - 2} \left( {\left( {L\left( {\tau \left( {H{}_{2}} \right) - \tau (\varGamma_{2} )} \right)} \right)} \right)} ds} \right], \\ \vdots \end{aligned} \right\}$$

## Results and discussion

In this section, we will apply the method presented in this paper to solve singular IVPs of Lane–Emden-type.

### *Example 1*

Consider the linear, homogeneous Lane–Emden differential equation

23 $$\varGamma^{{\prime \prime }} + \frac{2}{\tau }\varGamma^{{\prime }} - 2(2\tau^{2} + 3)\varGamma = 0,$$

with initial conditions

24 $$\varGamma (0) = 1\quad \varGamma^{{\prime }} (0) = 0.$$

Applying the Laplace transform on both sides, we get

25 $$\begin{aligned} & L(\tau \varGamma^{\prime \prime } ) + 2L(\varGamma^{\prime } ) - L(2(2\tau^{3} + 3\tau )\varGamma ) = 0, \\ & \quad \quad \quad - s^{2} L^{{\prime }} (\varGamma ) - 1 = L\{ 2(2\tau^{3} + 3\tau )\varGamma \} , \\ & L^{{\prime }} (\varGamma ) = - \frac{1}{{s^{2} }} - \frac{L}{{s^{2} }}\{ 2(2\tau^{3} + 3\tau )\varGamma \} . \\ \end{aligned}$$

By integrating both sides of Eq. (25) with respect to s, we have

$$\varGamma (s) = \int {L(\varGamma )ds} = - \int {s^{ - 2} } ds - \int {s^{ - 2} } L\left( {2(2\tau^{3} + 3\tau )\varGamma } \right)ds.$$

Taking Inverse Laplace transform on both sides, we get

$$\begin{aligned} L^{ - 1} (\varGamma (s)) & = - L^{ - 1} \left( {\int {s^{ - 2} ds} } \right) - \left( {\int {s^{ - 2} L\left( {2(2\tau^{3} + 3\tau )\varGamma } \right)} ds} \right), \\ \varGamma (\tau ) & = - L^{ - 1} \left( {\int {s^{ - 2} ds} } \right) - \left( {\int {s^{ - 2} L\left( {2(2\tau^{3} + 3\tau )\varGamma } \right)ds} } \right), \\ \varGamma (\tau ) & = 1 - L^{ - 1} \left( {\int {\frac{L}{{s^{2} }}(2(2\tau^{3} + 3\tau )\varGamma )ds} } \right). \\ \end{aligned}$$

Applying Homotopy perturbation method, we get a solution by an infinite series of components given by:

$$\varGamma (\tau ) = \sum\limits_{n = 0}^{\infty } {p^{n} } \varGamma_{n} (\tau ).$$

Thus Eq. (23) becomes

26 $$\sum\limits_{n = 0}^{\infty } {p^{n} } \varGamma_{n} (\tau ) = 1 - pL^{ - 1} \left( {\int {s^{ - 2} \left( {L\left( {\sum\limits_{n = 0}^{\infty } {p^{n} (2(2\tau^{3} + 3\tau )\varGamma_{n} (\tau )} } \right)ds} \right)} } \right).$$

Equating the coefficient of like power of p, we get

27 $$\begin{array}{*{20}l} {p^{0} :\varGamma_{0} = 1,} \hfill \\ {p^{1} :\varGamma_{1} = \tau^{2} + \frac{{\tau^{4} }}{5},} \hfill \\ {p^{2} :\varGamma_{2} = \frac{3}{10}\tau^{4} + \frac{13}{105}\tau^{6} + \frac{1}{90}\tau^{8} ,} \hfill \\ {p^{3} :\varGamma_{3} = \frac{3}{70}\tau^{6} + \frac{17}{630}\tau^{8} + \frac{59 \, }{11550}\tau^{10} + \frac{{\tau^{12} }}{3510},} \hfill \\ \begin{aligned} p^{4} :\varGamma_{4} : = \frac{{\tau^{8} }}{280} + \frac{{\tau^{10} }}{330} + \frac{{343\,\tau^{12} }}{386100} + \frac{{4987\tau^{14} }}{47297250} + \frac{{\tau^{16} }}{238680}, \hfill \\ \vdots \hfill \\ \end{aligned} \hfill \\ \end{array}$$

Thus we get the solution of this series as follows:

28 $$\begin{aligned} \varGamma & = \varGamma_{0} + \varGamma_{1} + \varGamma_{2} + \varGamma_{3} + \varGamma_{4} + \cdots , \\ & = 1 + \tau^{2} + \frac{{\tau^{4} }}{2} + \frac{{\tau^{6} }}{6} + \frac{{\tau^{8} }}{24} + \frac{{\tau^{10} }}{120} + \frac{{\tau^{12} }}{720} \cdots . \\ \end{aligned}$$

The closed form of the series (28) is $y = \exp (\tau^{2} )$ which gives an approximate solution of the problem.

**Fig. 1:**
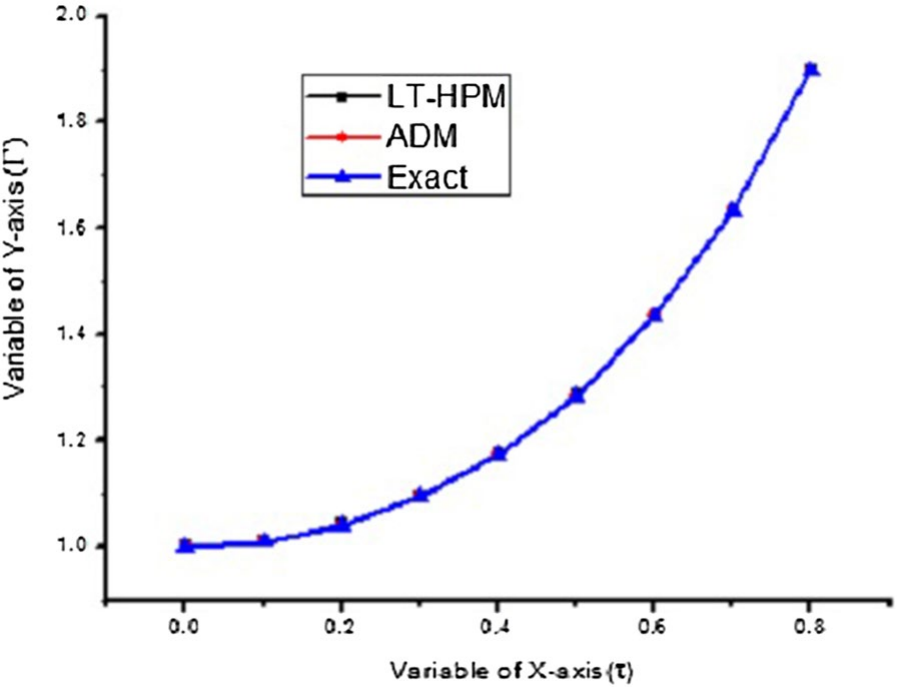
Comparison between LT-HPM, Exact and ADM solution

In this problem, the Table 1 shows the comparison of values of LT-HPM with exact and ADM in the terms of different values of $\tau$ (Fig. 1).

**Table 1:** The comparison with exact solution and ADM (Wazwaz 2001)

$\tau$	LT-HPM	ADM	Exact	Error (LT-HPM)	Error (ADM)
0.0	1	1	1	0.0	0.0
0.1	1.0100501670842	1.01005017	1.0100501670842	0.0	0.0
0.2	1.0408107741924	1.04081078	1.0408107741924	0.0	1E−9
0.3	1.0941742836956	1.09417428	1.0941742837052	9.6E−12	1E−9
0.4	1.1735108704484	1.17351087	1.1735108709918	5.434E−10	2.3E−8
0.5	1.2840254041884	1.28402542	1.2840254166877	1.24993E−8	3.5E−7
0.6	1.4333292517888	1.43332623	1.4333294145603	1.627715E−7	3.2E−6
0.7	1.6323147871342	1.63229556	1.6323162199554	1.4328212E−6	2.1E−5
0.8	1.8964714019044	1.89637596	1.8964808793049	9.99706E−4	1.1E−4

The graphical comparison of LT-HPM, exact solution and ADM solution are given as follows:

### *Example 2*

Consider the linear, homogeneous Lane–Emden differential equation

29 $$\varGamma^{\prime \prime } + \frac{2}{\tau }\varGamma^{\prime } + \varGamma^{n} = 0, \quad \tau \ge 0,\quad n = 0,1, \ldots,$$

subject to the initial condition

30 $$\varGamma (0) = 1,\quad \varGamma^{{\prime }} (0) = 0.$$

Taking Laplace transform on both sides, we get

31 $$\begin{aligned} & L(\tau \varGamma^{{\prime \prime }} ) + 2L(\varGamma^{{\prime }} ) + L(\tau \varGamma^{n} ) = 0, \\ & \quad - s^{2} L^{{\prime }} (\varGamma ) - \varGamma (0) + L(\tau \varGamma^{n} ) = 0, \\ \end{aligned}$$

putting n = 0 in Eq. (31), we get

$$\begin{aligned} & - s^{2} L^{{\prime }} (\varGamma ) - \varGamma (0) + L(\tau ) = 0. \\ & L^{{\prime }} (\tau ) = - \frac{1}{{s^{2} }} + \frac{1}{{s^{4} }}. \\ \end{aligned}$$

Integrating above equation with respect to s, we get

$$L(\tau ) = \int {( - s^{ - 2} + } s^{ - 4} )ds.$$

Taking inverse Laplace Transform on both sides, we get

$$\varGamma (\tau ) = L^{ - 1} \int {\left( { - \frac{1}{{s^{2} }} + \frac{1}{{s^{4} }}} \right)ds} ,$$

$$\varGamma (\tau ) = 1 - \frac{{\tau^{2} }}{6}.$$

which is the exact solution.

When n = 1 in Eq. (31), we get

$$- s^{2} L^{{\prime }} (\varGamma ) - \varGamma (0) + L(\tau \varGamma ) = 0.$$

$$L^{{\prime }} (\varGamma ) = - \frac{1}{{s^{2} }} + \frac{1}{{s^{2} }}L(\tau \varGamma ) = 0$$

Integrating above equation with respect to s, we get

$$L(\varGamma (\tau )) = - \int {s^{ - 2} } ds + \int {s^{ - 2} L(\tau \varGamma )ds}$$

Taking inverse Laplace transform on both sides, we get

$$\varGamma (\tau ) = - L^{ - 1} \left( {\int {s^{ - 2} } ds} \right) + L^{ - 1} \left( {\int {s^{ - 2} L(\tau \varGamma )ds} } \right),$$

$$\varGamma (\tau ) = 1 + L^{ - 1} \left( {\int {s^{ - 2} L(\tau \varGamma )ds} } \right).$$

Applying Homotopy Perturbation Method on both sides, we get

$$\sum\limits_{n = 0}^{\infty } {p^{n} \varGamma_{n} } (\tau ) = 1 + pL^{ - 1} \left( {\int {s^{ - 2} \left( {\sum\limits_{n = 0}^{\infty } {p^{n} \varGamma_{n} (\tau )} } \right)} ds} \right).$$

Equating the coefficient of like power of p, we get

$$\begin{array}{*{20}l} {p^{0} :\varGamma_{0} = 1,} \hfill \\ {p^{1} :\varGamma_{1} = - L^{ - 1} \left( {\int {s^{ - 2} L(\varGamma_{0} )ds} } \right) = - \frac{{\tau^{2} }}{6},} \hfill \\ {p^{2} :\varGamma_{2} = - L^{ - 1} \left( {\int {s^{ - 2} L(\varGamma_{1} )ds} } \right) = \frac{{\tau^{4} }}{5 \times 4!},} \hfill \\ {p^{3} :\varGamma_{3} = - L^{ - 1} \left( {\int {s^{ - 2} L(\varGamma_{2} )ds} } \right) = - \frac{{\tau^{6} }}{7 \times 6!},} \hfill \\ \begin{aligned} p^{4} :\varGamma_{4} = - L^{ - 1} \left( {\int {s^{ - 2} L(\varGamma_{3} )ds} } \right) = \frac{{\tau^{8} }}{9 \times 8!}, \hfill \\ \vdots \hfill \\ \end{aligned} \hfill \\ \end{array}$$

Thus we get the solution of this series as follows:

32 $$\begin{aligned} \varGamma & = \varGamma_{0} + \varGamma_{1} + \varGamma_{2} + \varGamma_{3} + \varGamma_{4} + \cdots . \\ & = 1 - \frac{{\tau^{2} }}{6} + \frac{{\tau^{4} }}{5 \times 4!} - \frac{{\tau^{6} }}{7 \times 6!} + \frac{{\tau^{8} }}{9 \times 8!} + \cdots . \\ \end{aligned}$$

The closed form of the series (32) is $\varGamma (\tau ) = \frac{\sin \tau }{\tau }$ which gives an exact solution of the problem.

The Table 2 shows the comparison of values of LT-HPM within the terms of different values of $\tau$.

**Table 2:** Comparison with exact solution

$\tau$	LT-HPM	Exact	Error (LT-HPM)
0.1	0.9983341665	0.998334166	−5E−10
0.2	0.993346654	0.993346654	−0E+0
0.3	0.9850673555	0.9850673555	−0E+0
0.4	0.9735458558	0.9735458558	−0E+0
0.5	0.9588510772	0.9588510772	−0E+0
0.6	0.9410707891	0.941070789	−1E−10
0.7	0.9203109825	0.9203109818	−7E−10
0.8	0.8966951163	0.8966951136	−2.7E−9
0.9	0.8703632416	0.8703632329	−8.7E−9
1.0	0.8414710097	0.8414709848	−2.49E−8

We established the graphical comparison of LT-HPM and exact solution as follows:

### *Example 3*

Consider the linear, non-homogenous Lane–Emden equation

33 $$\varGamma^{\prime \prime } + \frac{2}{\tau }\varGamma^{\prime } + \varGamma = 6 + 12\tau + \tau^{2} + \tau^{3}, \quad 0 \le \tau \le 2,$$

subject to initial conditions,

34 $$\varGamma (0) = 0,\quad \varGamma^{{\prime }} (0) = 0.$$

Applying Laplace transform on both sides, we get

$$\begin{aligned} & L(\tau \varGamma^{{\prime \prime }} ) + 2L(\varGamma^{{\prime }} ) + L(\tau \varGamma ) = L(6\tau + 12\tau^{2} + \tau^{3} + \tau^{4} ), \\ & L^{{\prime }} (\varGamma ) = s^{ - 2} L(\tau \varGamma ) - s^{ - 2} L\{ 6\tau + 12\tau^{2} + \tau^{3} + \tau^{4} \} . \\ \end{aligned}$$

By integrating above equation with respect to s, we get

$$L(\varGamma ) = - \int {s^{ - 2} L\left( {6\tau + 12\tau^{2} + \tau^{3} + \tau^{4} } \right)ds} + \int {s^{ - 2} L\left( {\tau \varGamma } \right)ds} .$$

Applying Inverse Laplace transform on both sides, we get

$$\varGamma (\tau ) = - L^{ - 1} \left( {\int { - s^{ - 2} L(6\tau + 12\tau^{2} + \tau^{3} + \tau^{4} )} ds} \right) + L^{ - 1} \left( {\int {s^{ - 2} L(\tau \varGamma )} ds} \right).$$

Applying Homotopy Perturbation Method, we get

35 $$\sum\limits_{n = 0}^{\infty } {p^{n} } \varGamma_{n} = \tau^{2} + \tau^{3\,} + \frac{{\tau^{4} }}{20} + \frac{{\tau^{5} }}{30} + pL^{ - 1} \left( {\int {s^{ - 2} L\left( {\sum\limits_{n = 0}^{\infty } {p^{n} \varGamma_{n} } (\tau )} \right)} ds} \right).$$

equating the coefficient of like power of p, we get

$$\begin{array}{*{20}l} {p^{0} :\varGamma_{0} = \tau^{2} + \tau^{3} + \frac{{\tau^{4} }}{20} + \frac{{\tau^{5} }}{30},} \hfill \\ {p^{1} :\varGamma_{1} = L^{ - 1} \left( {\int {s^{ - 2} \left( {L(\tau \varGamma_{0} (\tau ))} \right)} ds} \right) = - \frac{{\tau^{4} }}{20} - \frac{{\tau^{5} }}{30} - \frac{{\tau^{6} }}{840} - \frac{{\tau^{7} }}{1680},} \hfill \\ {p^{2} :\varGamma_{2} = L^{ - 1} \left( {\int {s^{ - 2} \left( {L(\tau \varGamma_{1} (\tau ))} \right)} ds} \right) = \frac{{\tau^{6} }}{840} + \frac{{\tau^{7} }}{1680} + \frac{{\tau^{8} }}{60480} + \frac{{\tau^{9} }}{151200},} \hfill \\ p^{3} :\varGamma_{3} = L^{ - 1} \left( {\int {s^{ - 2} \left( {L(\tau \varGamma_{2} (\tau ))} \right)} ds} \right) = - \frac{{\tau^{8} }}{60480} - \frac{{\tau^{9} }}{151200} - \frac{{\tau^{10} }}{6652800} - \frac{{\tau^{11} }}{19958400}, \hfill \\ p^{4} :\varGamma_{4} = L^{ - 1} \left( {\int {\left( {L(\tau \varGamma_{3} (\tau ))} \right)} ds} \right) = \frac{{\tau^{10} }}{6652800} + \frac{{\tau^{11} }}{19958400} + \frac{{\tau^{12} }}{1037836800} + \frac{{\tau^{13} }}{363235600}, \hfill \\ \hfill \\ \vdots \hfill \\ \end{array}$$

Thus we get the solution of this series as follows:

36 $$\begin{aligned} \varGamma & = \varGamma_{0} + \varGamma_{1} + \varGamma_{2} + \varGamma_{3} + \varGamma_{4} + \cdots . \\ \varGamma & = \tau^{2} + \tau^{3} . \\ \end{aligned}$$

The closed form of the series (36) is $\varGamma (\tau ) = \tau^{2} + \tau^{3}$ which gives an exact solution of the problem.

The comparison with exact solution is given by Table 3.

**Table 3:** The comparison with the exact solution

$\tau$	LT-HPM	Exact
0.0	0.0	0.0
0.1	0.011	0.011
0.2	0.048	0.048
0.3	0.117	0.117
0.4	0.224	0.224
0.5	0.375	0.375
0.6	0.576	0.576
0.7	0.833	0.833
0.8	1.152	1.152
0.9	1.539	1.539
1.0	2	2

The Table 3 shows the comparison of values of LT-HPM with exact in the terms of different values of $\tau$.

### *Example 4*

Consider the non-linear, homogenous Lane–Emden equation

37 $$\varGamma^{{\prime \prime }} + \frac{2}{\tau }\varGamma^{{\prime }} + \varGamma^{3} - (6 + \tau^{6} ) = 0\quad \tau \ge 0,$$

subject to the initial condition

38 $$\varGamma (0) = 0,\quad \varGamma^{{\prime }} (0) = 0.$$

Applying Laplace transform on both sides, we

$$\begin{aligned} & L(\tau \varGamma^{{\prime \prime }} ) + 2L(\varGamma^{{\prime }} ) + L(\tau \varGamma^{3} ) - L(6\tau + \tau^{7} ) = 0, \\ & \quad - s^{2} L^{{\prime }} (\varGamma ) = L(6\tau + \tau^{7} ) - L(\tau \varGamma^{3} ), \\ & L^{{\prime }} (\varGamma ) = - s^{ - 2} L(6\tau + \tau^{7} ) + s^{ - 2} L(\tau \varGamma^{3} ), \\ & L(\varGamma (\tau )) = - \int {s^{ - 2} L(6\tau + \tau^{7} )ds} + \int {s^{ - 2} L(\tau \varGamma^{3} )ds} , \\ \end{aligned}$$

Applying Inverse Laplace Transformation, we get

$$\varGamma (\tau ) = - L^{ - 1} \left( {\int {s^{ - 2} \left( {L(\tau \varGamma^{3} )} \right)} ds} \right) + L^{ - 1} \left( {\int {s^{ - 2} L(6\tau + \tau^{7} )ds} } \right).$$

Applying HPM both side, we get

39 $$\sum\limits_{n = 0}^{\infty } {p^{n} \varGamma_{n} } (\tau ) = \tau^{2} + \frac{{\tau^{8} }}{72} + pL^{ - 1} \left( {\int {s^{ - 2} L} \left( {\sum\limits_{n = 0}^{\infty } {p^{n} } H_{n} (\varGamma )} \right)} \right).$$

Equating the coefficient of like power of p, we get

$$\begin{aligned} p^{0} :\varGamma_{0} (\tau ) & = \tau^{2} + \frac{{\tau^{8} }}{72}, \\ p^{1} :\varGamma_{1} (\tau ) & = \int {s^{ - 2} L} \left( {\tau H_{0} (\varGamma )} \right) = - \frac{{\tau^{8} }}{72} - \frac{{\tau^{14} }}{5040} - \frac{{\tau^{20} }}{725760} - \frac{{\tau^{26} }}{262020096} \\ p^{2} :\varGamma_{2} (\tau ) & = \int {s^{ - 2} L} \left( {\tau H_{1} (\varGamma )} \right) = \frac{{\tau^{14} }}{5040} + \frac{{53\tau^{20} }}{12700800} + \frac{{25\tau^{26} }}{611380224} + \frac{{ \, 67 \, \tau^{32} }}{293462507520 \, } \\ & \quad + \frac{{ \, 491 \, \tau^{38} }}{652367154216960 \, } - \frac{{ \, \tau^{44} }}{896486037258240 \, } \\ p^{3} :\varGamma_{3} (\tau ) & = \int {s^{ - 2} L} \left( {\tau H_{2} (\varGamma )} \right) = - \frac{{71\tau^{20} }}{25401600} - \frac{{8173\tau^{26} }}{106991539200} - \frac{{37369 \, \tau^{32} }}{37661021798400} \\ & \quad - \frac{{ \, 72109 \, \tau^{38} }}{9133140159037440 \, } - \frac{{ \, 4514651 \, \tau^{44} }}{108501705089364787200 \, } \\ & \quad - \frac{{ \, 52841339\tau^{50} }}{368905797303840276480000 \, } - \frac{{276053\tau^{56} }}{1068698394448207408005120 \, } \\ & \quad + \frac{{\tau^{62} }}{101136362276346837073920}, \\ \end{aligned}$$

$$\begin{aligned} p^{4} :\varGamma_{4} (\tau ) & = \int {s^{ - 2} L} \left( {\tau H_{3} (\varGamma )} \right) = \frac{{12619\tau^{26} }}{320974617600} + \frac{{49591\tau^{32} }}{37661021798400} + \frac{{124658383 \, \tau^{38} }}{5860431602049024000} \\ & \quad + \frac{{ \, 11319804851 \, \tau^{44} }}{52216445574256803840000 \, } - \frac{{ \, 19811411311 \, \tau^{50} }}{12911702905634409676800000 \, } \\ & \quad + \frac{{ \, 575925932801\tau^{56} }}{74185480214613064239022080000} \\ & \quad + \frac{{ \, 3395344051061\tau^{68} }}{56214318223818043730058301931520000 \, } \\ & \quad + \frac{{636488953519\tau^{70} }}{9988541976464570807364056730289966153728000000} \\ & \quad - \frac{{298992611\tau^{74} \, }}{5632426047399250357186524610560000 \, } \\ & \quad + \frac{{18703\tau^{80} }}{397496390291087651691073162444800}, \\ \vdots \\ \end{aligned}$$

Thus we get the solution of this series as follows

$$\varGamma = \varGamma_{0} + \varGamma_{1} + \varGamma_{2} + \varGamma_{3} + \varGamma_{4} + \cdots \,\,.$$

40 $$\varGamma = \tau^{2} .$$

The closed form of the series (40), is $\varGamma (\tau ) = \tau^{2}$ which gives an exact solution of the problem.

The comparison with exact solution is given by Table 4.

**Table 4:** The comparison with exact solution

$\tau$	LT-HPM	Exact
0.0	0.0	0.0
0.1	0.1	0.1
0.2	0.4	0.4
0.3	0.9	0.9
0.4	0.16	0.16
0.5	0.25	0.25
0.6	0.36	0.36
0.7	0.49	0.49
0.8	0.64	0.64
0.9	0.81	0.81
1.0	1.0	1.0

The Table 4 shows the comparison of values of LT-HPM with exact in the terms of different values of $\tau$.

### *Example 5*

Consider the linear homogeneous differential equation

41 $$\varGamma^{{\prime \prime }} + \frac{2}{\tau }\varGamma^{{\prime }} + e^{\varGamma } = 0,$$

with the initial conditions

42 $$\varGamma (0) = 0,\quad \varGamma^{{\prime }} (0) = 0.$$

Applying the Laplace transform on both sides, we get

$$\begin{aligned} & L(\tau \varGamma^{{\prime \prime }} ) + 2\varGamma^{{\prime }} + L(\tau e^{\varGamma } ) = 0, \\ & \quad - s^{2} L^{{\prime }} (\varGamma ) = L(\tau e^{\varGamma } ), \\ & L^{{\prime }} (\varGamma ) = - s^{ - 2} L(\tau e^{\varGamma } ). \\ \end{aligned}$$

By integrating both sides with respect to s, we get

$$L(\varGamma ) = - \int {s^{ - 2} L(\tau e^{\varGamma } )ds} .$$

Applying inverse Laplace Transformation on both sides, we get

$$\varGamma (\tau ) = - L^{ - 1} \int {s^{ - 2} \left( {L(\tau e^{\varGamma } )} \right)} ds.$$

Applying HPM on both sides, we get

43 $$\sum\limits_{n = 0}^{\infty } {p^{n} \varGamma_{n} } (\tau ) = - pL^{ - 1} \left( {\left( {\sum\limits_{n = 0}^{\infty } {\int {s^{ - 2} } \left( {L(\tau e^{\varGamma } )} \right)} } \right)} \right)ds.$$

Equating the coefficient of like power of p, we get

44 $$\begin{aligned} & p^{0} :\varGamma_{0} (\tau ) = 0, \\ & p^{1} :\varGamma_{1} (\tau ) = - \frac{{\tau^{2} }}{3!}, \\ & p^{2} :\varGamma_{2} (\tau ) = \frac{{\tau^{4} }}{5!}, \\ & p^{3} :\varGamma_{3} (\tau ) = - \frac{{\tau^{6} }}{7!}, \\ & p^{4} :\varGamma_{4} (\tau ) = \frac{{\tau^{8} }}{9!}, \\ & \vdots \\ \end{aligned}$$

Thus we get the solution of this series as follows

45 $$\begin{aligned} \varGamma & = \varGamma_{0} + \varGamma_{1} + \varGamma_{2} + \varGamma_{3} + \varGamma_{4} + \cdots . \\ & = - \frac{{\tau^{2} }}{3!} + \frac{{\tau^{4} }}{5!} - \frac{{\tau^{6} }}{7!} + \frac{{\tau^{8} }}{9!} - \cdots . \\ \end{aligned}$$

### *Example 6*

Consider the nonlinear, homogeneous Lane–Emden differential equation

46 $$\varGamma^{{\prime \prime }} + \frac{2}{\tau }\varGamma^{{\prime }} + 4(2e^{\left( \varGamma \right)} + e^{{\left( {\frac{\varGamma }{2}} \right)}} ) = 0,\quad 0 \le \tau \le 1,$$

subject to initial conditions,

47 $$\varGamma (0) = 0,\quad \varGamma^{'} (0) = 0.$$

The exact solution of the problem is

48 $$\varGamma (\tau ) = - 2In(1 + \tau^{2} ).$$

Applying Laplace transform on both sides, we get

$$\begin{aligned} & L(\tau \varGamma^{{\prime \prime }} ) + 2L(\varGamma^{{\prime }} ) + L\left( {4\left( {2\tau e^{(\varGamma )} + \tau e^{{\left( {\frac{\varGamma }{2}} \right)}} } \right)} \right) = 0, \\ & \quad - s^{2} L^{{\prime }} (\varGamma ) + 4L\left( {2\tau e^{(\varGamma )} + \tau e^{{\left( {\frac{\varGamma }{2}} \right)}} } \right) = 0, \\ & L^{{\prime }} (\varGamma ) = s^{ - 2} 4\left( {L\left( {2\tau e^{(\varGamma )} + \tau e^{{\left( {\frac{\varGamma }{2}} \right)}} } \right)} \right). \\ \end{aligned}$$

Integrating the above equation with respect to s, we get

$$\varGamma (s) = \int {s^{ - 2} L(4(2\tau e^{(\varGamma )} + \tau e^{{\left( {\frac{\varGamma }{2}} \right)}} ))ds} .\,\,\,\,\,\,$$

Applying Inverse Laplace Transformation on both sides, we get

$$\varGamma = L^{ - 1} \left( {\int {s^{ - 2} \left( {L(4\tau \varGamma^{3} )} \right)} ds} \right).$$

Applying HPM on both sides, we get

49 $$\sum\limits_{n = 0}^{\infty } {p^{n} } \varGamma_{n} (\tau ) = pL^{ - 1} \left( {\int {s^{ - 2} } L\left( {\sum\limits_{n = 0}^{\infty } {p^{n} (4\tau H_{n} } (\varGamma ))ds} \right)} \right).$$

where $N(\varGamma ) = (2e^{(\varGamma )} + e^{{\left( {\frac{\varGamma }{2}} \right)}} )$ is the nonlinear operator, $H_{n} (\varGamma )$ is the He’s polynomial. Which is given by

$$\left. {\begin{array}{*{20}l} {H_{0} (\varGamma ) = \left( {2e^{{(\varGamma_{0} )}} + e^{{\left( {\frac{{\varGamma_{0} }}{2}} \right)}} } \right),} \hfill \\ {H_{1} (\varGamma ) = \varGamma_{1} \left( {2e^{{(\varGamma_{1} )}} + \frac{1}{2}e^{{\left( {\frac{{\varGamma_{1} }}{2}} \right)}} } \right),} \hfill \\ {H_{2} (\varGamma ) = \varGamma_{2} \left( {2e^{{(\varGamma_{1} )}} + \frac{1}{2}e^{{\left( {\frac{{\varGamma_{1} }}{2}} \right)}} } \right) + \frac{{\varGamma_{1}^{2} }}{2!}\left( {2e^{{(\varGamma_{1} )}} + \frac{1}{4}e^{{\left( {\frac{{\varGamma_{1} }}{2}} \right)}} } \right),} \hfill \\ \end{array} } \right\}$$

50 $$\left. \begin{aligned} & H_{3} (\varGamma ) = \varGamma_{3} \left( {2e^{{(\varGamma_{1} )}} + \frac{1}{2}e^{{\left( {\frac{{\varGamma_{1} }}{2}} \right)}} } \right) + \varGamma_{1} \varGamma_{2} \left( {2e^{{(\varGamma_{1} )}} + \frac{1}{4}e^{{\left( {\frac{{\varGamma_{1} }}{2}} \right)}} } \right) + \frac{{\varGamma_{1}^{3} }}{3!}\left( {2e^{{(\varGamma_{1} )}} + \frac{1}{8}e^{{\left( {\frac{{\varGamma_{1} }}{2}} \right)}} } \right), \\ & \vdots \\ \end{aligned} \right\}$$

using the value of Eq. (50) in Eq. (49) and equating the coefficient of like the power of p, we get

$$\left. {\begin{array}{*{20}l} {p^{0} :\varGamma_{0} = 0,} \hfill \\ {p^{1} :\varGamma_{1} = \left( {\int {s^{ - 2} \left( {L\left( {4\tau (H_{0} )} \right)} \right)} ds} \right) = - 2\tau^{2} ,} \hfill \\ {p^{2} :\varGamma_{2} = \left( {\int {s^{ - 2} \left( {L\left( {4\tau (H_{1} )} \right)} \right)} ds} \right) = \tau^{4} ,} \hfill \\ \end{array} } \right\}$$

51 $$\left. {\begin{array}{*{20}l} {p^{3} :\varGamma_{3} = \left( {\int {s^{ - 2} \left( {L\left( {4\tau (H_{2} )} \right)} \right)} ds} \right) = - \frac{2}{3}\tau^{6} ,} \hfill \\ {p^{4} :\varGamma_{4} = \left( {\int {s^{ - 2} \left( {L\left( {4\tau (H_{3} )} \right)} \right)} ds} \right) = \frac{1}{2}\tau^{8} ,} \hfill \\ \vdots \hfill \\ \end{array} } \right\}$$

Thus we get the solution of this series as follows:

52 $$\begin{aligned} \varGamma & = \varGamma_{0} + \varGamma_{1} + \varGamma_{2} + \varGamma_{3} + \varGamma_{4} + \cdots . \\ \varGamma (\tau ) & = - 2\left( {\tau^{2} - \frac{1}{2}\tau^{4} + \frac{1}{3}\tau^{6} - \frac{1}{4}\tau^{8} + \cdots .} \right) \\ \end{aligned}$$

The closed form of the series (52) is $\varGamma (\tau ) = - 2In(1 + \tau^{2} )$ which gives an exact solution of the problem.

### *Example 7*

Consider the nonlinear, homogeneous Lane–Emden differential equation

53 $$\varGamma^{{\prime \prime }} + \frac{2}{\tau }\varGamma^{{\prime }} - 6\varGamma = 4\varGamma In\varGamma ,\quad 0 \le \tau \le 1,$$

subject to the initial condition,

54 $$\varGamma (0) = 1,\quad \varGamma (0) = 0.$$

The exact solution of the equation is

55 $$\varGamma (\tau ) = e^{{\tau^{2} }} .$$

Applying Laplace Transform on both sides, we get

$$\begin{aligned} & L(\tau \varGamma^{{\prime \prime }} ) + L(2\varGamma^{{\prime }} ) - L(6\tau \varGamma ) - L(4\tau (\varGamma In\varGamma )) = 0, \\ & \quad - s^{2} L^{{\prime }} (\varGamma ) - 1 = L\{ 6\tau \varGamma + 4\varGamma \tau In\varGamma \} , \\ \end{aligned}$$

$$L^{{\prime }} (\varGamma ) = - \frac{1}{{s^{2} }} - \frac{1}{{s^{2} }}L\{ 6\tau \varGamma + 4\varGamma \tau In\varGamma \} .$$

Taking integration on both sides of above equation, we get

$$L(\varGamma ) = - \int {s^{ - 2} } ds - \int {s^{ - 2} L\{ 6\tau \varGamma + 4\varGamma \tau In\varGamma \} ds} .$$

Applying Inverse Laplace transform on both sides, we get

$$\varGamma (\tau ) = 1 - L^{ - 1} \left( {\int {s^{ - 2} L\left( {6\tau \varGamma + 4\varGamma \tau In\varGamma } \right)} ds} \right).$$

Applying HPM on both sides, we get

56 $$\sum\limits_{n = 0}^{\infty } {p^{n} \varGamma_{n} } (\tau ) = 1 - pL^{ - 1} \left( {\int {s^{ - 2} L\left( {\sum\limits_{n = 0}^{\infty } {p^{n} \varGamma_{n} (\tau )} } \right)} ds} \right).$$

Equating the coefficient of like power of p, we get

$$\begin{aligned} & p^{0} :\varGamma_{0} (\tau ) = 1, \\ & p^{1} :\varGamma_{1} (\tau ) = - \int {s^{ - 2} L\left( {6\tau \varGamma_{0} + 4\varGamma_{0} (\tau In\varGamma_{0} )} \right)} ds = \tau^{2} , \\ & p^{2} :\varGamma_{2} (\tau ) = - \int {s^{ - 2} L\left( {6\tau \varGamma_{1} + 4\varGamma_{1} (\tau In\varGamma_{1} )} \right)} ds = \frac{1}{2!}\tau^{4} , \\ & p^{3} :\varGamma_{3} (\tau ) = - \int {s^{ - 2} L\left( {6\tau \varGamma_{2} + 4\varGamma_{2} (\tau In\varGamma_{2} )} \right)} ds = \frac{1}{3!}\tau^{6} , \\ & & p^{4} :\varGamma_{4} (\tau ) = - \int {s^{ - 2} L\left( {6\tau \varGamma_{3} + 4\varGamma_{3} (\tau In\varGamma_{3} )} \right)} ds = \frac{1}{4!}\tau^{8} , \\ & p^{5} :\varGamma_{5} (\tau ) = - \int {s^{ - 2} L\left( {6\tau \varGamma_{4} + 4\varGamma_{4} (\tau In\varGamma_{4} )} \right)} ds = \frac{1}{5!}\tau^{10} , \\ & \vdots \\ \end{aligned}$$

Thus we get the solution of this series as follows:

$$\begin{aligned} \varGamma & = \varGamma_{0} + \varGamma_{1} + \varGamma_{2} + \varGamma_{3} + \varGamma_{4} + \cdots \cdots \\ & = 1 + \tau^{2} + \frac{1}{2!}\tau^{4} + \frac{1}{3!}\tau^{6} + \frac{1}{4!}\tau^{8} + \frac{1}{5!}\tau^{10} + \cdots . \\ \end{aligned}$$

57 $$\varGamma (\tau ) = \exp (\tau^{2} ).$$

The closed form of the series (57) is $\varGamma (\tau ) = \exp (\tau^{2} )$ which gives an exact solution of the problem.

The Table 5 shows the comparison of values of LT-HPM with exact in the terms of different values of $\tau$.

**Table 5:** The comparison with exact solution

$\tau$	LT-HPM	Exact	Error
0.0	1	1	0.0
0.1	1.010050167	1.010050167	0.0
0.2	1.040810773	1.040810774	1E−9
0.3	1.094174234	1.094174284	5E–8
0.4	1.173509973	1.173510871	8.98E−7
0.5	1.284016927	1.284025417	8.49E−6
0.6	1.43327584	1.433329415	0.19904038
0.7	1.632060167	1.63231622	2.56053E−4
0.8	1.895481173	1.896480879	9.99706E−4
0.9	2.359156039	2.247907987	−0.111248052
1.0	2.708333333	2.718281828	0.009948495

### *Example 8*

Consider the following Lane–Emden type differential equation:

58 $$\varGamma^{{\prime \prime }} + \frac{2}{\tau }\varGamma^{{\prime }} + \sin (\varGamma ) = 0,$$

subject to the initial condition

59 $$\varGamma (0) = 1,\quad \varGamma^{{\prime }} (0) = 0.$$

Applying Laplace Transform (LT) on both sides, we get

$$\begin{aligned} L(\tau \varGamma^{{\prime \prime }} ) + 2\varGamma^{{\prime }} + L(\tau \sin (\varGamma )) = 0, \hfill \\ - s^{2} L^{{\prime }} (\varGamma ) - 1 = - L(\tau \sin (\varGamma )), \hfill \\ L^{{\prime }} (\varGamma ) = - \frac{1}{{s^{2} }} + \frac{1}{{s^{2} }}\left( {L(\tau \sin (\varGamma ))} \right), \hfill \\ \end{aligned}$$

Integrating the above equation with respect to s, we get

$$\varGamma (s) = - \int {s^{ - 2} } ds + \int {s^{ - 2} } \left( {\left( {L(\tau \sin (\varGamma ))} \right)} \right)ds.$$

Applying ILT on both sides, we get

$$\varGamma (\tau ) = 1 + L^{ - 1} \left( {\int {s^{ - 2} } \left( {\left( {L(\tau \sin (\varGamma ))} \right)} \right)ds} \right).$$

Applying HPM on both sides, we get

60 $$\sum\limits_{n = 0}^{\infty } {p^{n} } \varGamma_{n} (\tau ) = 1 - pL^{ - 1} \left( {\int {s^{ - 2} } \left( {\sum\limits_{n = 0}^{\infty } {\left( {L(\tau H_{n} (\varGamma ))} \right)} } \right)ds} \right).$$

Here $\zeta (\varGamma ) = \sin (\varGamma )$ is a non-linear term and $H_{n} (\varGamma )$ is He’s polynomial.

$$\begin{aligned} H_{0} & = \sin \varGamma_{0} , \\ H_{1} & = \varGamma_{1} \cos \varGamma_{0} , \\ \end{aligned}$$

$$\begin{aligned} & H_{2} = - \left( {\frac{{\varGamma_{1}^{2} }}{2}} \right)\sin \varGamma_{0} + \varGamma_{2} \cos \varGamma_{0} , \\ & H_{3} = - \left( {\frac{{\varGamma_{1}^{3} }}{6}} \right)\cos \varGamma_{0} - \varGamma_{1} \varGamma_{2} \sin \varGamma_{0} + \varGamma_{3} \cos \varGamma_{0} , \\ & \vdots \\ \end{aligned}$$

Equating the coefficients like power of p, we get

$$\begin{aligned} & p^{0} :\varGamma_{0} = 1, \\ & p^{1} :\varGamma_{1} = L^{ - 1} \left\{ {\int {s^{ - 2} L\left( {\tau H_{0} } \right)} ds} \right\} = - \frac{{\tau^{2} }}{6}k_{1} , \\ & p^{2} :\varGamma_{2} = L^{ - 1} \left\{ {\int {s^{ - 2} L\left( {\tau H_{1} } \right)} ds} \right\} = \frac{1}{120}k_{1} k_{2} \tau^{4} , \\ & p^{3} :\varGamma_{3} = L^{ - 1} \left\{ {\int {s^{ - 2} L\left( {\tau H_{2} } \right)} ds} \right\} = k_{1} \left( {\frac{1}{3024}k_{1}^{2} - \frac{1}{5040}k_{1} k_{2}^{2} } \right)\tau^{6} , \\ & p^{4} :\varGamma_{4} = L^{ - 1} \left\{ {\int {s^{ - 2} L\left( {\tau H_{3} } \right)} ds} \right\} = k_{1} k_{2} \left( {\frac{ - 107}{3265920}k_{1}^{2} + \frac{1}{362880}k_{2}^{2} } \right)x^{8} , \\ & \vdots \\ \end{aligned}$$

Thus we get the solution of this series as follows:

$$\begin{aligned} \varGamma & = \varGamma_{0} + \varGamma_{1} + \varGamma_{2} + \varGamma_{3} + \varGamma_{4} + \cdots \\ \varGamma & = 1 - \frac{{\tau^{2} }}{6}k_{1} + \frac{1}{120}k_{1} k_{2} \tau^{4} + k_{1} \left( {\frac{1}{3024}k_{1}^{2} - \frac{1}{5040}k_{1} k_{2}^{2} } \right)\tau^{6} \\ & \quad + k_{1} k_{2} \left( {\frac{ - 107}{3265920}k_{1}^{2} + \frac{1}{362880}k_{2}^{2} } \right)x^{8} + \cdots (61) \\ \end{aligned}$$

where

$$k_{1} = \sin (1),\quad k_{2} = \cos (1),$$

## Conclusions

In this communication, we have successfully employed the Homotopy Perturbation Method with Laplace Transform (LT-HPM) to obtain exact solutions for singular IVPs of Lane–Emden-type equations. We also find the accuracy of this method which gives us very attractive results in the terms of power series. This method can accelerate the rapid convergence of series solution when compared with Homotopy Perturbation Method using Laplace Transform. Very recently, the LT-HPM has been extensively applicable in many fields of science and engineering to solve these types of problems because of its dependability and the attenuation in the size of computations. The graphical representation of such types of problems shows that the LT-HPM is a promising tool for singular IVP’s of Lane–Emden type, and in some cases, yields exact solutions in two iterations.
